# Functional apoptosis profiling identifies MCL-1 and BCL-xL as prognostic markers and therapeutic targets in advanced thymomas and thymic carcinomas

**DOI:** 10.1186/s12916-021-02158-3

**Published:** 2021-11-16

**Authors:** Denise Müller, Paolo Mazzeo, Raphael Koch, Mark-Sebastian Bösherz, Stefan Welter, Alexander von Hammerstein-Equord, Marc Hinterthaner, Lucia Cordes, Djeda Belharazem, Alexander Marx, Philipp Ströbel, Stefan Küffer

**Affiliations:** 1 Institute of Pathology, University Medical Center Göttingen, University of Göttingen, Robert-Koch-Str. 40, 37075 Göttingen, Germany; 2 Department of Haematology and Medical Oncology, University Medical Centre Göttingen, Göttingen, Germany; 3 Thoracic Surgery Department, Lung Clinic Hemer, Hemer, Germany; 4 Department of Heart, Thoracic and Vascular Surgery, University Medical Center Göttingen, Göttingen, Germany; 5 Department of Thoracic and Cardiovascular Surgery, University Medical Center, Göttingen, Germany; 6 Institute of Pathology, University Medical Centre Mannheim and Medical Faculty Mannheim, Heidelberg University, Mannheim, Germany

**Keywords:** MCL-1, BCL-xL, BH3 mimetics, Thymoma, Thymic carcinoma

## Abstract

**Background:**

Multi-omics studies have shown a high and lack of common driver mutations in most thymomas (TH) and thymic carcinomas (TC) that hamper the development of novel treatment approaches. However, deregulation of apoptosis has been proposed as a common hallmark of TH and TC. BH3 profiling can be utilized to study the readiness of living cancer cells to undergo apoptosis and their dependency on pro-survival BCL-2 family proteins.

**Methods:**

We screened a cohort of 62 TH and TC patient samples for expression of BCL-2 family proteins and used the TC cell line 1889c and native TH for dynamic BH3 profiling and treatment with BH3 mimetics.

**Results:**

Immunohistochemical overexpression of MCL-1 and BCL-xL was a strong prognostic marker of TH and TC, and BH3 profiling indicated a strong dependency on MCL-1 and BCL-xL in TH. Single inhibition of MCL-1 resulted in increased binding of BIM to BCL-xL as an escape mechanism that the combined inhibition of both factors could overcome. Indeed, the inhibition of MCL-1 and BCL-xL in combination induced apoptosis in a caspase-dependent manner in untreated and MCL-1-resistant 1889c cells.

**Conclusion:**

TH and TC are exquisitely dependent on the pro-survival factors MCL-1 and BCL-xL, making them ideal candidates for co-inhibition by BH3 mimetics. Since TH show a heterogeneous dependency on BCL-2 family proteins, upfront BH3 profiling could select patients and tailor the optimal therapy with the least possible toxicity.

**Supplementary Information:**

The online version contains supplementary material available at 10.1186/s12916-021-02158-3.

## Background

Thymomas (TH) and thymic carcinomas (TC) are rare epithelial tumors of the thymus (TET). The World Health Organization (WHO) classifies TH into type A, AB, B1, B2, and B3 according to the morphology of the neoplastic epithelial cells and the proportion of immature lymphocytes [1]. TH show organotypic features, such as epithelial cells with cortical and medullary differentiation [2] and the capacity to promote the maturation of thymocytes. In contrast, TC have lost these features and are essentially indistinguishable from carcinomas in other organs.

Radical surgery is the only curative treatment, but advanced TH and TC often require a multimodal approach with radio-chemotherapy [3, 4]. Chemotherapy is the most common treatment for relapsed TET. Tyrosine kinase inhibitors such as sunitinib may be an option for relapsing TC [5, 6]. However, although the overall response rates are typically high [7, 8], most tumors eventually become refractory to these treatments.

The biology of TET is poorly understood [9–13]. Although recent multi-omics molecular studies revealed a few recurrent mutations, such as alterations of *GTF2I*, *HRAS*, and *NRAS* in TH, and rare mutations of epigenetic modifier genes in TC, TET are among the adult cancers with the lowest tumor mutational burden (TMB) and are not driven by mutations in common oncogenes [10, 14]. A significant obstacle to a better understanding of TET biology is the lack of suitable in vivo or in vitro tumor models. Thus, functional profiling of primary tissue samples could be the key to identifying novel vulnerabilities in TET. BH3 profiling is a tool that allows interrogating dependencies in the mitochondrial apoptosis pathway of freshly isolated viable tumor cells [15]. This assay measures the readiness of the cell to undergo apoptosis and their dependence on pro-survival BCL-2 family proteins. In brief, living cells are exposed to synthetic BH3 peptides that mimic the activity of endogenous pro-apoptotic proteins. By titration of either activator or sensitizer peptides, the level of mitochondrial apoptotic priming towards apoptosis can be quantified. The clinical availability of so-called BH3 mimetic drugs [16, 17] makes upfront dynamic BH3 profiling ideal for targeted therapeutic approaches and helps select patients and minimize cytotoxicity. The key regulators of the mitochondrial apoptosis pathway, the BCL-2 family proteins BCL-2, BCL-xL, BCL-w, and MCL-1, bind to BAK or BAX and prevent the oligomerization and pore formation at the outer mitochondrial membrane [18]. The BCL-2 family interaction network reprogramming is closely related to cancer survival and therapeutic resistance [19, 20]. The inhibition of single anti-apoptotic factors like MCL-1, BCL-2, and BCL-xL was successfully used in leukemia [21–23] and was proposed in several solid cancers [24–26]. Apoptosis-related genes, including *BIRC3*, *NOXA*, *MTCH2*, and *cFlip*, have already been proposed to be involved in tumor progression and drug resistance, especially in B3 TH and in TC [27, 28]. In the healthy thymus of mice, MCL-1 is required for the survival of mature cortical and medullary thymic epithelial cells and the maintenance of the thymic architecture [29]. Previous studies described a copy number gain of the *MCL-1* gene locus on chromosome 1q in about 51% of all subtypes with increased frequencies in B3 (70%) and TC (57%), as well as copy number gains of the *BCL-2* gene locus (18q21.33) in up to 42% of all TC. High expression of BCL-2 also correlates with decreased survival and was suggested as a potential therapeutic target in advanced TH and TC [30].

In this study, we explored the relevance of pro-survival BCL-2 family proteins in TH and TC. Immunohistochemistry was used to measure the expression of TP53, BCL-2, MCL-1, BCL-xL, and NOXA in a set of 62 TH and TC with known clinical follow-up and to correlate the results with survival. BH3 profiling was used to investigate apoptotic priming and to directly measure the effect of inhibition of MCL-1 by AZD5991, BCL-xL by A-1331852, and BCL-2 by Venetoclax (ABT-199) in primary patient samples. We suggest that the specific inhibition of MCL-1 and BCL-xL alone or in combination may be a promising and realistic treatment option for selected TET patients.

## Methods

### Clinical patient data and tissues

Formalin-fixed, paraffin-embedded (FFPE) TH and TC tissue samples were classified according to the recent WHO classification 2015 of tumors of the thymus. The tumor stage was assessed according to the modified Masaoka-Koga classification [31] by PS and AM (Table 1). Native TET and squamous cell cancer of the lung (SCCL) were provided by the Thoracic Surgery Departments Hemer and University Medical Center Göttingen with informed patient consent (Additional file: Table S1). The ethics committee approved the collection and use of samples of the University Medical Center Göttingen (GÖ 912/15).

**Table 1:** Clinicopathological parameters of 62 TH and TC patients

Patients	62
Male (%)	28 (45)
Female (%)	34 (55)
Age (range)	61 (28–88)
Type (%)	
A	7 (11.3)
AB	19 (30.6)
B1	2 (3.2)
B2	16 (25.8)
B3	9 (14.5)
C	9 (14.5)
Masaoka-Koga stage (%)	
1	16 (25.8)
2	20 (32.2)
3	14 (22.6)
4	8 (12.9)
Unknown	4 (6.5)
Median follow-up time in months (range)	46 (1–240)
Reported death (%)	20 (32.3)

### Cell culture

The human TC cell line 1889c was kindly provided by Ehemann et al. [12]. The SCCL cell line HCC15 (ACC 496) was purchased from DSMZ-German Collection of Microorganisms and Cell Cultures GmbH. Cell lines were cultured in RPMI-1640 medium supplemented with 10% fetal bovine serum, 2mM L-glutamine, and 100U/ml penicillin/streptomycin (Gibco, USA) at 37°C in a 5% CO_2_ humidified environment. All chemical compounds used for experimental procedures are listed in Additional file: Table S2.

### Cell viability assay and IC50 generation

Cell viability was determined using CellTiter Glo One Solution Assay (Promega, USA) according to the manufacturer’s recommendations. Luminescence was measured using a Tecan Plate Reader 2000 (Tecan, Switzerland). Relative IC50 was generated within Prism 8 (GraphPad Software, LCC) by normalizing single measurements, log transform drug concentration, and performing a dose-response curve fitting. IC50 values were calculated from at least three biological replicates using nonlinear regression algorithms.

### Cell transfection with siRNA and expression plasmids

Cells were transfected with either plasmid DNA or siRNA. Plasmid transfection was performed using the X-tremeGENE HP DNA transfection reagent (Merck, Germany) according to the manufacturer’s instructions. In brief, 100μL transfection mix containing serum-free RPMI-1640 cell culture medium, 2μg plasmid DNA, and 4μl transfection reagent was incubated for 15min at room temperature and added to 4 × 10^5^ cells in 2-ml medium directly after seeding (Additional file: Table S3). For siRNAs, Lipofectamine RNAiMAX transfection reagent (Thermo Fisher, USA) was used according to the manufacturer’s protocol. Briefly, a transfection mix of 250μl serum-free RPMI-1640, 200nM siRNA, and 7.5μl transfection reagent was incubated for 5min and added to 4 × 10^5^ cells in 2-ml medium (Additional file: Table S3).

### Protein extraction and western blot

Protein isolation from cells and fresh frozen tumor samples were performed using RIPA lysis buffer containing 1x protease inhibitor cocktail cOmplete (Roche, Switzerland), 1mM PMSF, and 1mM orthovanadate (Sigma-Aldrich, USA). Protein concentration was determined using DC™ Protein Assay (Bio-Rad, Germany). Western blots were performed using precast Mini Protein TGX gels and the semi-dry Trans-Blot Turbo^TM^ System (Bio-Rad, Germany). Antibodies used for specific gene detection are shown in Additional file: Table S2.

### Immunoprecipitation (IP)

IP was performed using 400-μg whole-cell protein lysates isolated in 1ml RIPA lysis buffer. To reduce unspecific bead binding, lysates were pre-incubated with 50μl protein G sepharose beads (GE Healthcare, USA) for 2h followed by overnight incubation with 5μl anti-BIM antibody (Cell Signaling, USA) at 4°C. Protein G sepharose beads were added for a 2-h incubation at 4°C. Beads were sedimented and washed with PBS, and proteins were denatured and dissolved in 30μl Laemmli buffer (Bio-Rad, Germany) at 95°C for 5 min. Fifteen microliters of the samples was immunoblotted as described above.

### DNA isolation and quantitative PCR (qPCR)

DNA was isolated from a 10-μm FFPE tissue slice using the InnuPREP FFPE DNA Extraction Kit (Jena Analytic, Germany) according to the manufacturer’s instructions. DNA quantity was determined using the Qubit dsDNA HS Assay (Invitrogen, USA). qPCR was performed on a LightCyler 480 II (Roche, Switzerland) using the 2x qPCRBIO SyGreen Mix Lo-ROX Kit (PCR Biosystems, UK) with indicated primers (Additional file: Table S3). CT values of MCL-1 and BCL-xL were normalized to the stable chromosome 3p and non-amplified healthy thymus samples. Gene amplification was defined as the normalized value to be greater than the mean of healthy thymus samples plus two standard deviations.

### Isolation of single cells from primary tissue

Primary tissue samples of the thymus and lung were minced; washed with Organoid Wash Medium (OWM) containing Advanced DMEM/F12, 10mM HEPES, GlutaMAX, 100μg/ml Primocin (Thermo Fisher, USA), and 0.1% bovine serum albumin (Sigma-Aldrich, USA); and digested with Organoid Digestion Medium containing OWM with 0.1% Collagenase Crude Type XI (Sigma-Aldrich), 10.5μM Y-27632 (AdooQ Bioscience, USA), and 10μg/ml DNAseI (Sigma-Aldrich, USA). Cell suspensions were applied to a 100-μm filter to remove residual tissue and cell agglomerates. Erythrocytes were lysed using ACK Lysing Buffer (Gibco; USA). Cells were resuspended in RPMI-1640 cell culture medium.

### BH3 profiling

BH3 profiling was performed according to Koch et al. [32]. In order to reduce noise by lymphocytes and fibroblasts, epithelial cells were labeled with immunofluorescent E-cadherin (clone 67A4) (Thermo Fisher, USA) antibody for 1h before measurement. Cells were treated in a 384-well-plate with BH3 peptides using BIM at 10, 1, 0.3, 0.1, and 0.01μM; BAD and HRK at 80 and 8μM; MS1 at 10, 3, and 1μM; and PUMA and FS1 at 10μM. Treatment with the BIM peptide assesses the functionality of BAX and BAK. BAD binds and antagonizes BCL-2, BCL-xL, BCL-w, and BFL-1. While HRK specifically binds and antagonizes BCL-xL, MS1 binds and antagonizes MCL-1. Additionally, the BCL-2 inhibitor ABT-199, the MCL-1 inhibitor AZD5991, and the BCL-xL inhibitor A-1331852 were used at 0.25 and 1μM. Dimethyl sulfoxide (DMSO) was used as negative control and alamethicin (Ala) as a positive control. Intracellular cytochrome c was stained with an immunofluorescence-labeled antibody (clone 6H2.B4) (Biolegend, USA), and cells were subjected to flow cytometry. Relative cytochrome c release of E-cadherin-positive cells was assessed by 1 − [(sample-pos.ctrl.)/(neg.ctrl.-pos.ctrl.)].

### Immunohistochemistry

Multi-tissue arrays (TMA) containing two 1.5-mm representative punches of 62 TH and TC patient samples were used for immunohistochemical (IHC) stainings (Table 1). All antibodies were established on positive control tissues chosen from the Human Protein Atlas (http://www.proteinatlas.org). Stainings were performed on 2-μm sections according to a standard protocol on an Autostainer (Agilent, USA). In brief, antigen retrieval was performed at 95°C in pH 6 or pH 9 Envision FLEX target retrieval solution in a PT Link Module (Agilent, USA) followed by 1-h incubation with primary antibodies (Additional file: Table S2). Samples were washed with PBS and incubated with an appropriate secondary antibody (EnVision Flex+, Dako) for 30min. Two individual observers (DM and PS) evaluated stainings for both cores of a respective case and graded as positive when >50% of the tumor cells were positive. Staining intensity was scored 0 to 2 (0, negative staining; 1, weak staining; 2, strong staining). The average staining intensity of two cores was taken for further analysis. To evaluate the best clinical separation and to define the optimal threshold for dividing IHC low and high staining intensities, the cutoff finder was used as described by Budczies et al. [33]. These resulting cutoff values are given in the corresponding figure legends.

### Statistical analysis and data presentation

Statistical analysis was performed using Prism 8 (GraphPad Software, LCC). Kaplan-Meier curves were compared using the log-rank test. Significant differences between the two groups were calculated using either Student’s *t*-test or the Mann-Whitney *U* test depending on the normality of signal distribution, which was assessed by the Shapiro-Wilk test. Multiple *t*-tests were performed using one-way ANOVA. Bivariate correlations were carried out using Pearson (for continuous variables) or Spearman (for ordinal variables) correlation coefficients. Data are presented as mean ± standard error of the mean (SEM) unless stated otherwise. *p*-values were annotated as follows: *p* < 0.05 (*), *p* < 0.005 (**), and *p* < 0.0005 (***).

## Results

### Expression of MCL-1 and BCL-xL has prognostic relevance in TH and TC

**Fig. 1:**
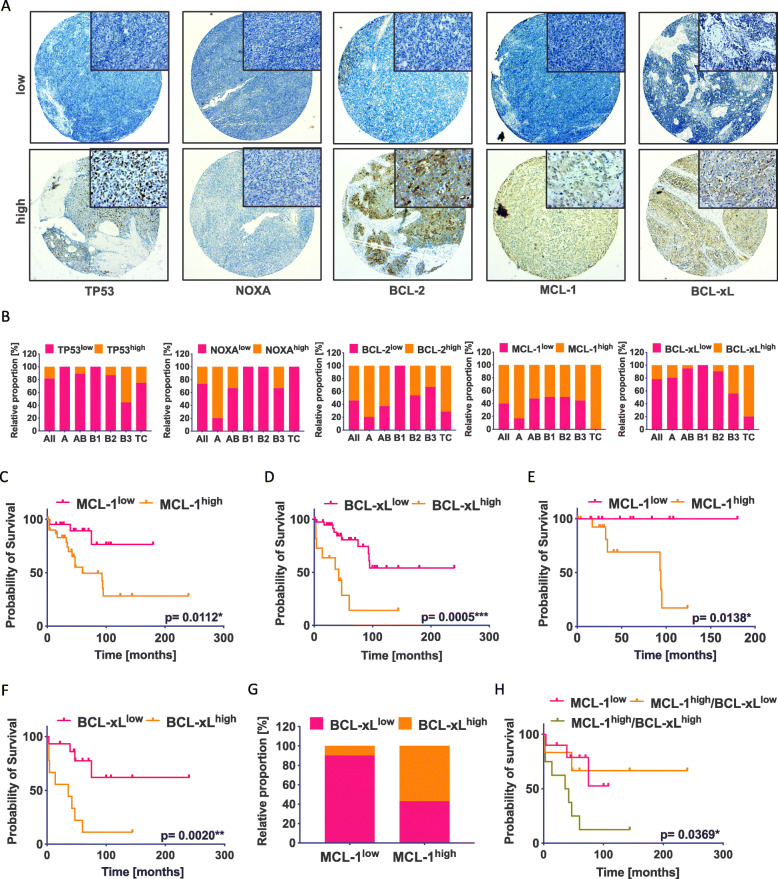
IHC stainings of MCL-1 and BCL-xL in TH and TC correlate with OS. **A** Representative images of IHC expression of TP53, NOXA, BCL-2, MCL-1, and BCL-xL and **B** the distribution of staining intensities in TET. **C** Kaplan-Meier analysis of all TH and TC showed a significant better OS for patients with low MCL-1 expression (cutoff = 0.875; *p* = 0.0112*) and **D** with low BCL-xL expression (cutoff = 0.875; *p* = 0.0005***). **E** Type A (*n* = 6), AB (*n* = 19), and B1 (*n* = 2) TH showed better OS for patients with low MCL-1 levels (*p* = 0.0138*) and **F** BCL-xL showed the strongest prognostic impact (*p*=0.0020**) in type B2 (*n* = 10) and B3 (*n* = 9) TH and TC (*n* = 5). **G** Correlation between BCL-xL and MCL-1 expression in type B2 (*n* = 10) and B3 (*n* = 9) TH and TC (*n* = 5). **H** Patients with strong expression of both MCL-1 and BCL-xL had the worst prognosis (*p* = 0.0369*)

To investigate the prognostic impact of the BCL-2 family proteins, we performed IHC stainings of TP53, BCL-2, MCL-1, BCL-xL, and the pro-apoptotic factor NOXA in 62 TH and TC with known clinical history (Table 1 and Fig. 1A). We observed a marked difference in the expression of single factors among the TH subtypes and TC (Fig. 1B). In only 60% of type B3 TH and 30% of TC, a strong expression of TP53 was observed and NOXA was expressed in 80% of type A TH but was not detectable in most other types. BCL-2 showed a strong expression in 85% of type A TH and in 80% of TC, followed by AB (65%), B2 (55%), and B3 (40%) but was absent in type B1 TH. MCL-1 was positive in 60% of type AB, B1, B2, and B3 TH; in 90% type A TH; and in all TC (100%). BCL-xL expression was frequent in type B3 TH (50%) and TC (90%). TH and TC with high expression of MCL-1 and BCL-xL showed a significantly shorter OS than tumors with low expression levels (Fig. 1C, D). However, no differences in survival were observed for the expression of TP53, NOXA, and BCL-2 (Additional file: Fig. S1A-C).

Since the histological subtype is linked to the inherent aggressiveness in TH, we next analyzed A, AB, and B1 TH and the more aggressive group of TH types B2 and B3 and TC separately. Interestingly, the prognosis seemed to be more affected by the expression level of MCL-1 in the type A, AB, and the B1 TH group (Fig. 1E), and by BCL-xL expression in type B2 and B3 TH and TC (Fig. 1F). Although MCL-1 did not show a significant difference in type B2 (*n* = 12) and B3 TH (*n* = 9) and TC (*n* = 5), there was a similar trend as for A, AB, and B1 TH (Additional file: Fig. S1D). All other factors analyzed did not result in any significant differences in either group (Additional file: Fig. S1E-I). When grouping B2 and B3 TH and TC according to MCL-1 and BCL-xL expression (Fig. 1G), we found a significantly decreased tumor survival in patients with high expression of both MCL-1 and BCL-xL (Fig. 1H). With only one case, the group of BCL-xL^high^ and MCL-1^low^ tumors was too small to be included.

### Inhibition of MCL-1 and BCL-xL induces apoptosis in the chemo-resistant TC cell line 1889c

**Fig. 2:**
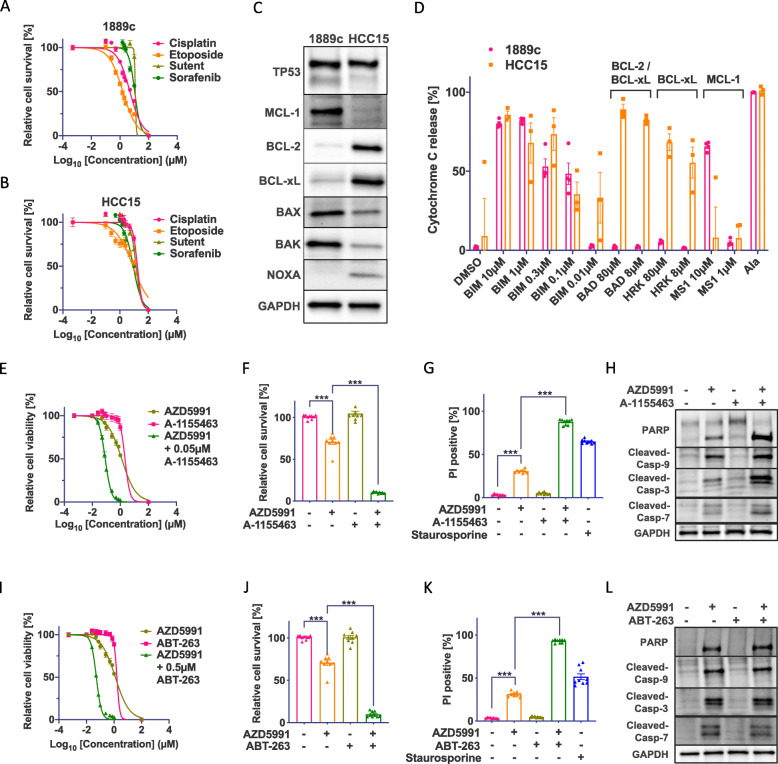
Analysis of BCL-2 family proteins in the chemo-resistant 1889c reveals an MCL-1-dependent apoptotic priming. **A** Dose-response curve of 1889c and **B** HCC15 for cisplatin and etoposide (0.1–10μM) and sunitinib and sorafenib (0.1–10μM). **C** Western blot analysis of TP53, MCL-1, BCL-2, BCL-xL, BAX, BAK, and NOXA in 1889c and HCC15. **D** BH3 profiling shows priming for MCL-1 in 1889c and BCL-2/BCL-xL in HCC15. **E** Dose-response curve of 1889c after a 24-h single treatment with AZD5991 and A-1155463 (0.01–1μM) and combined treatment with AZD5991 and A-1155463 (0.05μM). **F** Cell viability and **G** PI staining of 1889c after a 24-h single treatment with AZD5991 (0.5μM) and A-1155463 (0.05μM) and the combination of AZD5991 and A-1155463. **H** Western blot analysis of PARP cleavage and caspase activation of 1889c after a 6-h single treatment with AZD5991 (0.5μM) and A-1155463 (0.05μM) and the combination of AZD5991 and A-1155463. **I** Dose-response curve of 1889c after a 24-h single treatment with AZD5991, ABT-263, and AZD5991 (0.01–1μM) in combination with ABT-263 (0.5μM). **J** Cell viability and **K** PI staining of 1889c after a 24-h single treatment with AZD5991 (0.5μM) and ABT-263 (0.5μM) and the combination of AZD5991 and ABT-263. **L** Analysis of PARP cleavage and caspase activation of 1889c after a 6-h single treatment with AZD5991 (0.5μM) and ABT-263 (0.5μM) and the combination of AZD5991 and ABT-263 by western blot

First, we investigated the response to common first- and second-line therapeutics of TH and TC. For functional in vitro analyses, we used the *TP53* mutated TC cell line 1889c and the well-characterized SCCL cell line HCC15 [12, 34] (Additional file: Fig. S2A). Treatment with cisplatin, etoposide, sunitinib, and sorafenib revealed a strong resistance with IC50 concentrations between 17.3 and 1.6 μM (Fig. 2A, B and Additional file: Fig. S2B) [35].

Western blot analyses showed contrasting profiles with an abundance of MCL-1 and low levels of BCL-2, BCL-xL, and NOXA in 1889c and low levels of MCL-1 with high BCL-2 and BCL-xL levels in HCC15 (Fig. 2C). To investigate whether the protein expression correlated with the apoptotic priming, we performed BH3 profiling with the BH3-only proteins BIM, BAD, HRK, and MS1. While 1889c showed a dependency on MCL-1, HCC15 was primed for BCL-2/BCL-xL (Fig. 2D). To target individual pro-survival proteins, we next selected the most specific and clinically relevant MCL-1 inhibitor AZD5991, the specific BCL-xL inhibitor A-1155463, the BCL-2, and BCL-xL inhibitor ABT-263 (Navitoclax), and the specific BCL-2 inhibitor ABT-199 (Venetoclax). Cell viability of 1889c reduced by 50% on treatment with 1μM AZD5991 (Additional file: Fig. S2C, E, and F). As predicted, the cell viability of HCC15 showed a strong reduction upon treatment with A-1155463 and ABT-263 (Additional file: Fig. S2D, E, and G).

In line with previous reports that MCL-1 is linked to BCL-x L[36], the treatment of 1889c with AZD5991 in combination with either A-1155463 or ABT-263 showed a synergistic effect by decreasing the IC50 of AZD5991 by 15- to 24-fold (Fig. 2E, I, Additional file: Fig. S2H-J) and reduced cell viability down to 10% (Fig. 2F, J). PI staining confirmed 90% cell death (Fig. 2G, K). The combination with ABT-199 had no additional effect (Additional file: Fig. S2H and K). To evaluate apoptosis induction in 1889c, we performed western blot analyses. While single treatment with AZD5991 only partially activated caspase-3, caspase-7, caspase-9, and PARP, the combination of AZD5991 with either A-1155463 or ABT-263 showed a strong apoptosis induction (Fig. 2H, L).

To verify whether the MCL-1 and BCL-xL inhibition-induced apoptosis is solely caspase-dependent, we pretreated 1889c with the pan-caspase inhibitor zVAD-fmk. This rescued 80% of cells from a combined MCL-1 and BCL-xL inhibitory treatment (Additional file: Fig. S2L) and decreased caspase-3, caspase-7, and caspase-9 activation (Additional file: Fig. S2M). In contrast, zVAD-fmk was not able to rescue 1889c from caspase-independent apoptosis induced by staurosporine (Additional file: Fig. S2L) indicating an MCL-1- and BCL-xL-specific survival dependency of 1889c.

In summary, we could show that the inhibition of MCL-1 alone is not sufficient to induce apoptosis in 1889c, while the combined inhibition of MCL-1 and BCL-xL induces caspase-dependent cell death without any additional stimulation.

### Acquired resistance against MCL-1 inhibition is reversible by combined treatment with MCL-1 and BCL-xL inhibitors

**Fig. 3:**
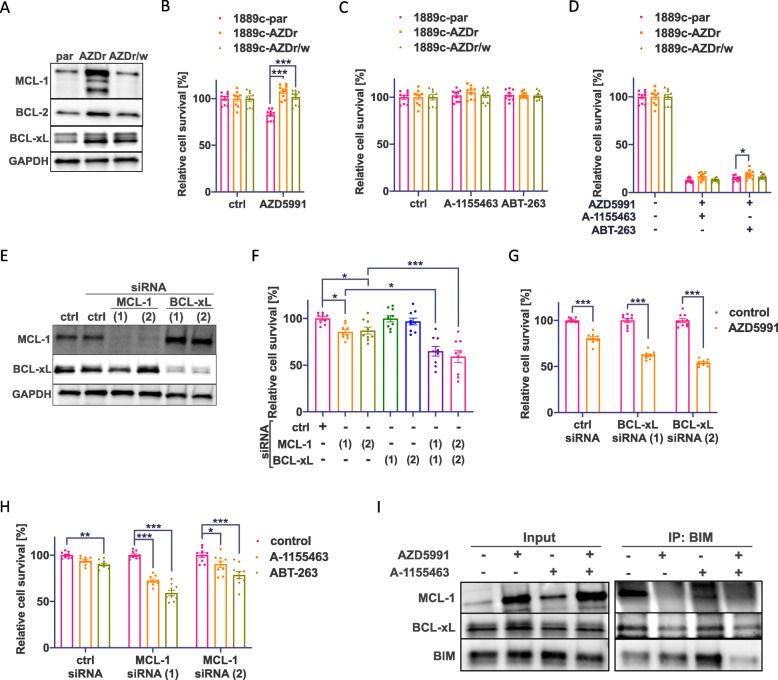
AZD5991 resistance induction, siRNA knockdown, and IP of BIM show a synergistic effect of MCL-1 and BCL-xL. **A** Western blot showing upregulation of MCL-1 and BCL-xL in AZD5991 resistant 1889c (1889c-AZDr). **B** Cell viability assays of 1889c, 1889c-AZDr, and resistant cells after cessation of treatment (1889c-AZDr/w) show partial reversibility of the induced AZD5991 resistance. **C** 1889c-AZDr and 1889c-AZDr/w show no increased sensitivity towards single treatment with A-1155463 (0.05μM) and ABT-263 (0.5μM) but **D** are sensitive towards a combinational treatment with AZD5991 (0.5μM). **E** Western blot analysis and **F** cell viability assay of 1889c after MCL-1 and BCL-xL knockdown with two individual siRNAs. **G** Cell viability assay of 1889c after BCL-xL knockdown and treatment with AZD5991 (0.5μM). **H** Cell viability assay of 1889c after MCL-1 knockdown and 24-h treatment with A-1155463 (0.05μM) or ABT-263 (0.5μM). **I** Redistribution of BIM in 1889c cells after 4-h treatment with AZD5991 (0.1μM), A-1155463 (0.1μM), or a combination of both (western blot after BIM IP)

Treatment resistance against MCL-1 inhibitors is frequently observed in AML [37]. We induced a treatment resistance towards MCL-1 inhibition by cultivating 1889c cells with increasing concentrations of AZD5991 (0.01–5μM) over 2 months. Resistant 1889c cells (1889c-AZDr) showed increased MCL-1, BCL-2, and BCL-xL protein expression (Fig. 3A). MCL-1 and BCL-2 expression levels were reversed back to baseline after 2 weeks of drug withdrawal (1889c-AZDr/w) (Fig. 3A). However, the resistance against AZD5991 was only partially lost (Fig. 3B). Interestingly, treatment of 1889c-AZDr or 1889c-AZDr/w cells with A-1155463 and ABT-263 targeting BCL-xL alone did not affect cell viability, while a combination of AZD5991 and either A-1155463 or ABT-263 showed a substantial reduction in cell viability in both lines (Fig. 3C, D). This indicated that an acquired resistance towards MCL-1 inhibition of 1889c is based on a synergistic effect between MCL-1 and BCL-xL.

### Single inhibitor treatment after siRNA knockdown of either MCL-1 or BCL-xL is inferior to combined inhibition

It has been reported that dual targeting of BCL-2 family proteins, especially in combination with BCL-xL inhibition, may cause thrombocytopenia [37, 38]. Since single treatment with MCL-1 inhibitors is effective in SCCL cell lines with high MCL-1 and low BCL-xL expression [39], we asked whether single BH3 mimetics directed against the most prevalent protein in a given TH or TC would be sufficient to induce anti-tumor activity. siRNA knockdown of either *MCL-1* or *BCL-xL* alone in 1889c cells resulted in a minor decrease in cell viability; however, double knockdown of MCL-1 and BCL-xL decreased cell viability to 60% (Fig. 3E, F). To mimic MCL-1^high^/BCL-xL^low^ tumors, we knocked down *BCL-xL* by siRNA followed by AZD5991 treatment. This resulted in a 50% reduction of cell viability (Fig. 3G). siRNA knockdown of *MCL-1* to mimic MCL-1^low^/BCL-xL^high^ tumors treated with A-1155463 or ABT-263 reduced cell viability down to 50% (Fig. 3H). This indicates that single-agent treatment in tumors expressing either MCL-1 or BCL-xL might not be sufficient to induce apoptosis and cell death to the same degree as combinational inhibition.

### Altered distribution of BIM during MCL-1 and BCL-xL inhibition

In AML, it was shown that the inhibition of BCL-2 increases the association of BIM to MCL-1, resulting in treatment resistance [40]. Besides, the distribution of BIM among BCL-2 proteins was described to favor BCL-2/BCL-xL co-dependencies in MCL-1 dependent myeloma cells [36]. We asked whether similar relocations of BIM occur during treatment with MCL-1 and/or BCL-xL inhibitors. IP of BIM in 1889c revealed a strong association between BIM and MCL-1 in untreated cells (Fig. 3I). Although the treatment with the MCL-1 inhibitor AZD5991 showed an overall increase in MCL-1 expression, it was dissociated from BIM. There was no difference in BIM bound to BCL-xL after A-1155463 treatment. However, a combination treatment again reduced the association of BIM to MCL-1. This indicated a more complex interaction of MCL-1 and BCL-xL as a pro-survival mechanism in 1889c cells.

### Loss of NOXA stabilizes MCL-1 but does not protect from MCL-1/BCL-xL co-targeting

**Fig. 4:**
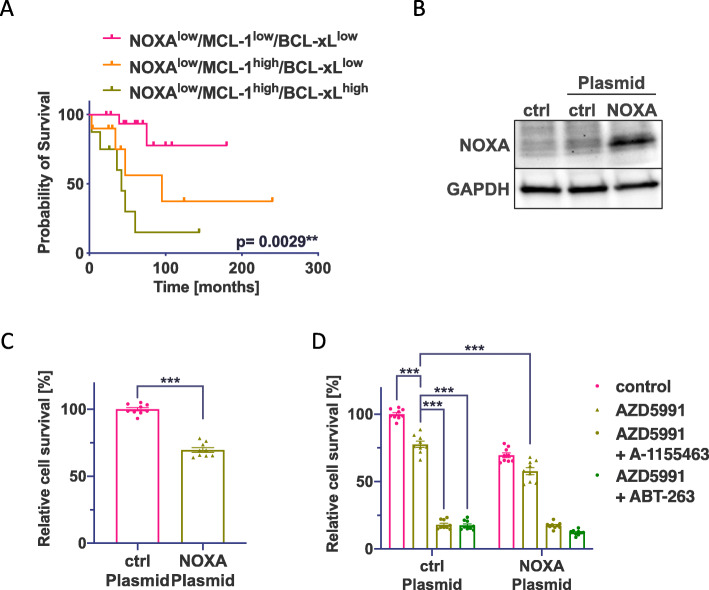
Correlation between IHC staining of MCL-1 and BCL-xL and OS in NOXA^low^ TH and TC and re-expression of NOXA in 1889c. **A** Kaplan-Meier analysis of NOXA^low^/MCL-1^low^/BCL-xL^low^ (*n* = 17), NOXA^low^/MCL-1^high^/BCL-xL^low^ (*n* = 10), and NOXA^low^/MCL-1^high^/BCL-xL^high^ (*n* = 8) resolve a significant better OS for patients with low NOXA signal depending on MCL-1 and BCL-xL signals (*p* = 0.0029*). **B** Western blot analysis of 1889c cells transfected with control or NOXA plasmid. **C** Cell viability assay of 1889c after induced expression of NOXA. **D** Cell viability assay of NOXA transfected 1889c cells after a 24-h single treatment with AZD5991 (0.5μM) or combined treatment with A-1155463 (0.05μM) or ABT-263 (0.5μM)

NOXA was highly expressed in type A TH but largely absent in all other subtypes and the expression level of NOXA alone had no statistical impact on clinical outcome (Additional file: Fig. S1C). However, when both NOXA and MCL-1 were considered, the OS of patients with NOXA^low^/MCL-1^high^/BCL-xL^high^ tumors was significantly shorter than that of patients with NOXA^low^/MCL-1^low^/BCL-xL^low^ tumors (Fig. 4A).

NOXA is an endogenous inhibitor of MCL-1, and loss of NOXA is described as a common mechanism leading to treatment resistance [41, 42]. We found that induced expression of NOXA reduced cell viability of 1889c by 30% (Fig. 4B, C). Besides, in cells with induced expression of NOXA, the additional inhibition of MCL-1 by AZD5991 further decreased cell viability by 20% (Fig. 4D). Combinational treatment using AZD5991 and A-1155463 or ABT-263 showed similar responses in both NOXA-deficient cells and in cells with induced NOXA expression (Fig. 4D). These data indicate that the absence of NOXA stabilizes MCL-1 and renders the cells more resistant to MCL-1 inhibitors.

### qPCR of the MCL-1 gene locus on chromosome 1q correlates with protein expression and OS

**Fig. 5:**
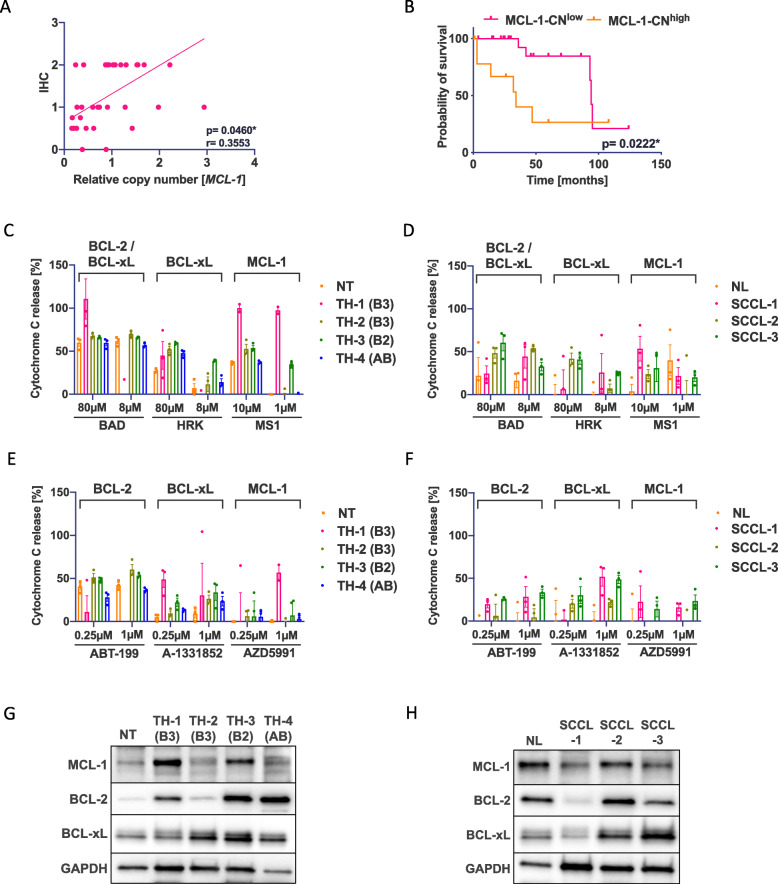
BH3 profiling of primary patient tissue samples predicts apoptotic priming and therapy response to BH3 mimetics. **A** Correlation between IHC MCL-1 staining intensity and relative gene copy number (CN) of *MCL*-1 (*n*=32, 5 A, 13 AB, 1 B1, 5 B2, 4 B3, and 4 TC) (*p*=0.0460*). **B** Significantly better overall survival for patients with low copy numbers of MCL-1 (MCL-1-CN^low^) (*p*=0.0222*). **C**, **D** BH3-profiling of a healthy thymus (NT), two type B3 (TH-1 and 2), a type B2 (TH-3), and a type AB (TH-4) TH revealed marked priming for MCL-1/BCL-xL in the type B3 TH. **E** Western blot analysis of MCL-1, BCL-2, and BCL-xL expression in the same tissue samples. **F**, **G** BH3 profiling and **H** western blot analysis of a healthy lung (NL) and three SCCL samples

Petrini et al. described a frequent amplification on chromosome 1q including the gene locus of *MCL-1* in type B2 and B3 TH and TC [30]. A less frequent amplification on chromosome 19 where the *BCL-xL* gene is located was also observed. We wondered whether the amplification of either gene correlated with protein expression and clinical prognosis in TH and TC. *MCL-1* amplification was found in 38% of all samples with the highest frequency in type B2 (80%) and B3 TH (75%) and TC (50%). *MCL-1* gene amplification showed a moderate correlation with MCL-1 protein expression detected by IHC (Fig. 5A) and was significantly correlated to OS (Fig. 5B). We also found *BCL-xL* amplified in 50% of all tumor samples, but we did not find a significant correlation to expression or OS (Additional file: Fig. S3A and B).

### BH3 profiling predicts apoptotic priming in clinical TET samples

BH3 profiling of ex vivo cancer cells is already in use to predict BCL-2 dependency in hematopoietic cancers [32, 43]. We wanted to investigate whether pharmacological BH3 profiling can be applied to primary TET patient tissue. We performed BH3 profiling of single cells from native tissue specimens including one healthy thymus, one type AB, one type B2, and two type B3 TH in comparison to one healthy lung tissue and three SCCL samples (Additional file: Table S1 and Additional file: Fig. S3C-K). To decrease noise from possibly primed lymphocytes, cytochrome C release was only measured in E-cadherin-positive cells. Healthy thymus and TH showed a higher and constant dependency on BCL-2/BCL-xL (Fig. 5C) in comparison to lung tissues (Fig. 5D). One type B3 TH (TH-1) showed specific MCL-1 priming (1μM MS1) and the type B2 TH (TH-3) showed a dependency for MCL-1 (1μM MS1) and BCL-xL (8μM HRK). We then measured cytochrome c release after treatment with ABT-199, A-1331852, and AZD5991 (Fig. 5E, F). Except for TH-1, all samples showed a good response to BCL-2 inhibition. The MCL-1 primed TH-1 showed the best response to AZD5991 and A-1331852 in TET (Fig. 5D). This was also reflected by a strong expression of MCL-1 and a moderate expression of BCL-2 and BCL-xL on western blot (Fig. 5G). Although the healthy lung and the SCCL samples showed a strong expression of single anti-apoptotic factors (Fig. 5F), a lower and more heterogeneous apoptotic priming and no specific apoptotic dependency were found (Fig. 5F, H). These data indicate that BH3 profiling is a powerful tool to predict the apoptotic priming and potential response to BH3 mimetics of clinical TH and TC tissue samples.

## Discussion

The virtual absence of common driver mutations in TH and TC [10] points to alternative oncogenic mechanisms in the pathogenesis of TET, but the lack of suitable cellular or animal models has hampered the search for new treatment options in these tumors. Suppression of apoptosis, e.g., through upregulation of pro-survival proteins, is one of the most important hallmarks of cancer [19]. In this context, functional BH3 profiling is an extremely useful tool to identify tumor types that depend on targetable anti-apoptotic BCL2 family members like BCL-xL and MCL-1 for their survival. Importantly, BH3 profiling interrogates ex vivo isolated viable tumor cells for their readiness to undergo apoptosis or dependence on pro-survival BCL-2 family proteins and is, therefore, an ideal bridge for tumors such as TET where no cellular models are available. Previous studies have already shown altered expression of apoptosis-related factors such as BIRC3, NOXA, MTCH2, and cFlip in TH and TC [27, 28, 44] and that the inhibition of BCL-2 family proteins, including MCL-1 and BCL-xL, induces apoptosis in TET cell lines [30].

We here confirm previous observations by Petrini et al. [30] that many TH and TC show amplification of the *MCL-1* gene locus on chromosome 1q. Our findings extend these data by showing that TET belong to the growing family of solid tumors that depend critically on MCL-1 and BCL-xL for survival and chemotherapy resistance. We believe that these observations could and should be exploited for therapeutic purposes with BH3 mimetics, but the heterogeneity of these tumors will make upfront BH3 profiling mandatory for patient selection. Furthermore, our findings indicate that any clinical trial using BH3 mimetics in TET should ideally address both MCL-1 and BCL-xL.

MCL-1 is necessary for the maintenance of cortical and medullary thymic epithelial cells [10, 29], suggesting that it may also be relevant for TET development. The earliest study by Chen et al. showed frequent co-expression of MCL-1 and BCL-2 in many aggressive TET [45]. Our IHC analysis of 62 TET samples with known clinical follow-up identified MCL-1 and BCL-xL as prognostic markers. Notwithstanding the inherent limitations of a retrospective study based on relatively small case numbers, we found that the combination of strong MCL-1 and BCL-xL expression together with low levels of NOXA was particularly informative to identify patients with poor OS. In line with a previous report by Petrini et al. [30], overexpression of MCL-1 correlated with increased copy numbers of the *MCL-1* gene. As pointed out previously [30], the MCL-1 copy number alteration is probably the result of gains of the whole 1q arm rather than focal *MCL-1* gene amplification. The gain of 1q has long been recognized as a cytogenetic hallmark of aggressive TET [46].

Treatment with single agents like Venetoclax has been successfully used in hematopoietic cancers [32, 37, 40] but was less effective in solid tumors, including breast cancer, SCCL, and melanoma cell lines [38, 47, 48]. However, the situation in melanoma resembles TET since MCL-1 and BCL-xL are considered the main pro-survival factors [38]. Single inhibition of either factor in melanoma cells did not have a significant effect, and only the combined inhibition resulted in the induction of apoptosis. In the same line, we found strong priming for MCL-1 in the chemo-resistant TC cell line 1889c using dynamic BH3 profiling. Only the combination of the MCL-1 inhibitor AZD5991 with either A-1155463 or ABT-263 but not a single treatment induced a potent caspase-dependent apoptosis and almost entirely eliminated 1889c in a nanomolar range. The siRNA knockdown of MCL-1 and/or BCL-xL supports the model that a certain threshold of pro-survival proteins must be inhibited to induce apoptosis and that other BCL-2 family members sequester pro-apoptotic proteins [49]. It has been observed that the single inhibition of BCL-2/BCL-xL leads to treatment resistance by increased MCL-1 or BCL-xL levels and redistribution of BIM [36, 40]. Our results show that BIM was sequestered away from MCL-1 and shifted to BCL-xL as an escape mechanism in 1889c treated with AZD5991. These findings indicate that in most cases the inhibition of MCL-1 alone will not be sufficient to induce satisfactory clinical effects in a therapeutic setting due to a dynamic synergy with BCL-xL as proposed before [50].

Thrombocytopenia or liver damage as a specific side effect of BCL-xL inhibitors [17, 49] makes combinational treatment difficult. However, He et al. recently described an interesting approach to circumvent BCL-xL inhibition toxicity by a BCL-xL degrader with a better anti-tumor activity [51].

Importantly, BH3 profiling of primary human tissue samples revealed strong priming for MCL-1 and/or BCL-xL in malignant type B2 and B3 TH, but not in SCCL. In line with these measurements, only TH but not SCCL samples showed cytochrome c release after treatment with the inhibitors ABT-199, A1331852, and AZD5991.

## Conclusions

In summary, these findings indicate that TET are exquisitely dependent on pro-survival factors such as MCL-1 and BCL-xL and that suppression of apoptosis is a major functional hallmark of these non-oncogenic driven tumors. This observation makes TET ideal candidates for clinical trials with BH3 mimetics that ideally address both MCL-1 and BCL-xL. Given the observed marked heterogeneity of TET concerning their dependence on pro-survival BCL-2 family proteins, upfront BH3 profiling should be used to select those patients with the most significant benefit and to tailor the optimal therapy with the least possible toxicity in a targeted therapeutic approach.

## Supplementary Information

**Additional file 1: Figure S1.** Survival analysis of IHC staining of BCL-2 family proteins in TH and TC. (A-C) Kaplan-Meier analysis of TP53 (cutoff = 0.25), BCL-2 (cutoff = 0.125), and NOXA (cutoff = 1.25), for all subtypes did not show a significant impact on patient OS, (D) of low vs. high MCL-1 in type B2 (n=12) and B3 (n=9) TH and TC (n=5), (E) BCL-2 in type A (n=5), AB (n=19), and B1 (n=2) TH, (F) BCL-2 in type B2 (n=13) and B3 (n=9) TH and TC (n=7), (G) TP53 in type B2 (n=15) and B3 (n=9) TH and TC (n=8), (H) NOXA in type A (n=5), AB (n=18) and B1 (n=2) TH and (I) NOXA in type B2 (n=10) and B3 (n=9) TH and TC (n=5) did not show any significant impact on patient OS. **Figure S2.** Analysis of BCL-2 family members and specific inhibitors in 1889c and HCC15. (A) Sanger sequencing revealed a *TP53* mutation (c.738G>T, p.M246I) in 1889c. (B) IC50 concentrations of 1889c and HCC15 after a single treatment with Cisplatin and Etoposide (0.1-10μM, 72h) and Sunitinib and Sorafenib (0.1-10μM, 48h). (C, D) Dose-response curve and (E) IC50 concentrations of 1889c and HCC15 after a single treatment with AZD5991, A-1155463, ABT-199, and ABT-263 (0.01-1μM, 24h). Relative cell survival of (F) 1889c and (G) HCC15 after a 24h single treatment with AZD5991, A-1155463, ABT-199, and ABT-263. (H) IC50 concentrations of 1889c after a 24h combined treatment with AZD5991 (0.01-1μM) and A-1155463 (0.01μM, 0.025μM, 0.05μM), ABT-199 (0.01μM, 0.025μM, 0.05μM) and ABT-263 (0.1μM, 0.25μM, 0.5μM). (I) Dose-response matrix of 1889c for the combinational treatment with AZD5991 and A-1155463 and (J) for AZD5991 and ABT-263. (K) IC50 curves of 1889c after a 24h single treatment with AZD5991 and ABT-199 (0.01-1μM) and the combination of AZD5991 with ABT-199 (0.05μM). (L) Cell viability assay and (M) Western Blot analysis of 1889c cells treated for 24h with AZD5991 (0.5μM), A-1155463 (0.05μM), ABT-263 (0.5μM), and staurosporine (0.25μM) alone or after 3h pretreatment with the pan-caspase inhibitor zVAD-fmk (100μM). **Figure S3.** qPCR analysis and BH3 profiling of patient tissue. (A) Correlation analysis of IHC staining of BCL-xL with the relative copy number of *BCL-xL* (n=32, 5 A, 13 AB, 1 B1, 5 B2, 4 B3, and 4 TC). (B) Kaplan-Meier analysis showed no significant impact of *BCL-xL* copy number on patient OS. (C-G) BH3-profiling of primary patient tissue of healthy thymus (NT), two type B3 (TH-1 and 2), one type B2 (TH-3), and one type AB (TH-4) TH. (H-K) BH3-profiling of primary patient tissue of healthy lung (NL) and three SCCL samples. **Table S1.** Clinicopathological parameters of primary patient tissue samples. **Table S2.** Antibodies and chemical compounds. **Table S3.** Primer sequences, plasmids, and siRNAs.

**Additional file 2.** Unprocessed Fig. 2C.

## Data Availability

The datasets supporting the conclusions of this article are included within the article and its supplementary files.

## Abbreviations

ANOVA: Analysis of variance
BH3: B-cell lymphoma 2 (Bcl-2) homology 3
FFPE: Formalin-fixed and paraffin-embedded
OS: Overall survival
SCCL: Small cell cancer of the lung
siRNA: Small interfering RNA
TC: Thymic carcinoma
TET: Thymic epithelial tumors
TH: Thymoma
