# Supplementary material for: Functional apoptosis profiling identifies MCL-1 and BCL-xL as prognostic markers and therapeutic targets in advanced thymomas and thymic carcinomas
Source: BMC Med. 2021 Nov 16;19:300. doi: 10.1186/s12916-021-02158-3 (PMC8594228; doi:10.1186/s12916-021-02158-3)
Supplement: Supplementary file 1 — Additional file 1: Figure S1. Survival analysis of IHC staining of BCL-2 family proteins in TH and TC. (A-C) Kaplan-Meier analysis of TP53 (cutoff = 0.25), BCL-2 (cutoff = 0.125), and NOXA (cutoff = 1.25), for all subtypes did not show a significant impact on patient OS, (D) of low vs. high MCL-1 in type B2 (n=12) and B3 (n=9) TH and TC (n=5), (E) BCL-2 in type A (n=5), AB (n=19), and B1 (n=2) TH, (F) BCL-2 in type B2 (n=13) and B3 (n=9) TH and TC (n=7), (G) TP53 in type B2 (n=15) and B3 (n=9) TH and TC (n=8), (H) NOXA in type A (n=5), AB (n=18) and B1 (n=2) TH and (I) NOXA in type B2 (n=10) and B3 (n=9) TH and TC (n=5) did not show any significant impact on patient OS. Figure S2. Analysis of BCL-2 family members and specific inhibitors in 1889c and HCC15. (A) Sanger sequencing revealed a TP53 mutation (c.738G>T, p.M246I) in 1889c. (B) IC50 concentrations of 1889c and HCC15 after a single treatment with Cisplatin and Etoposide (0.1-10μM, 72h) and Sunitinib and Sorafenib (0.1-10μM, 48h). (C, D) Dose-response curve and (E) IC50 concentrations of 1889c and HCC15 after a single treatment with AZD5991, A-1155463, ABT-199, and ABT-263 (0.01-1μM, 24h). Relative cell survival of (F) 1889c and (G) HCC15 after a 24h single treatment with AZD5991, A-1155463, ABT-199, and ABT-263. (H) IC50 concentrations of 1889c after a 24h combined treatment with AZD5991 (0.01-1μM) and A-1155463 (0.01μM, 0.025μM, 0.05μM), ABT-199 (0.01μM, 0.025μM, 0.05μM) and ABT-263 (0.1μM, 0.25μM, 0.5μM). (I) Dose-response matrix of 1889c for the combinational treatment with AZD5991 and A-1155463 and (J) for AZD5991 and ABT-263. (K) IC50 curves of 1889c after a 24h single treatment with AZD5991 and ABT-199 (0.01-1μM) and the combination of AZD5991 with ABT-199 (0.05μM). (L) Cell viability assay and (M) Western Blot analysis of 1889c cells treated for 24h with AZD5991 (0.5μM), A-1155463 (0.05μM), ABT-263 (0.5μM), and staurosporine (0.25μM) alone or after 3h pretreatment with the pan-caspase inhibitor zVAD-fm [file 12916_2021_2158_MOESM1_ESM.pdf]

Figure S1

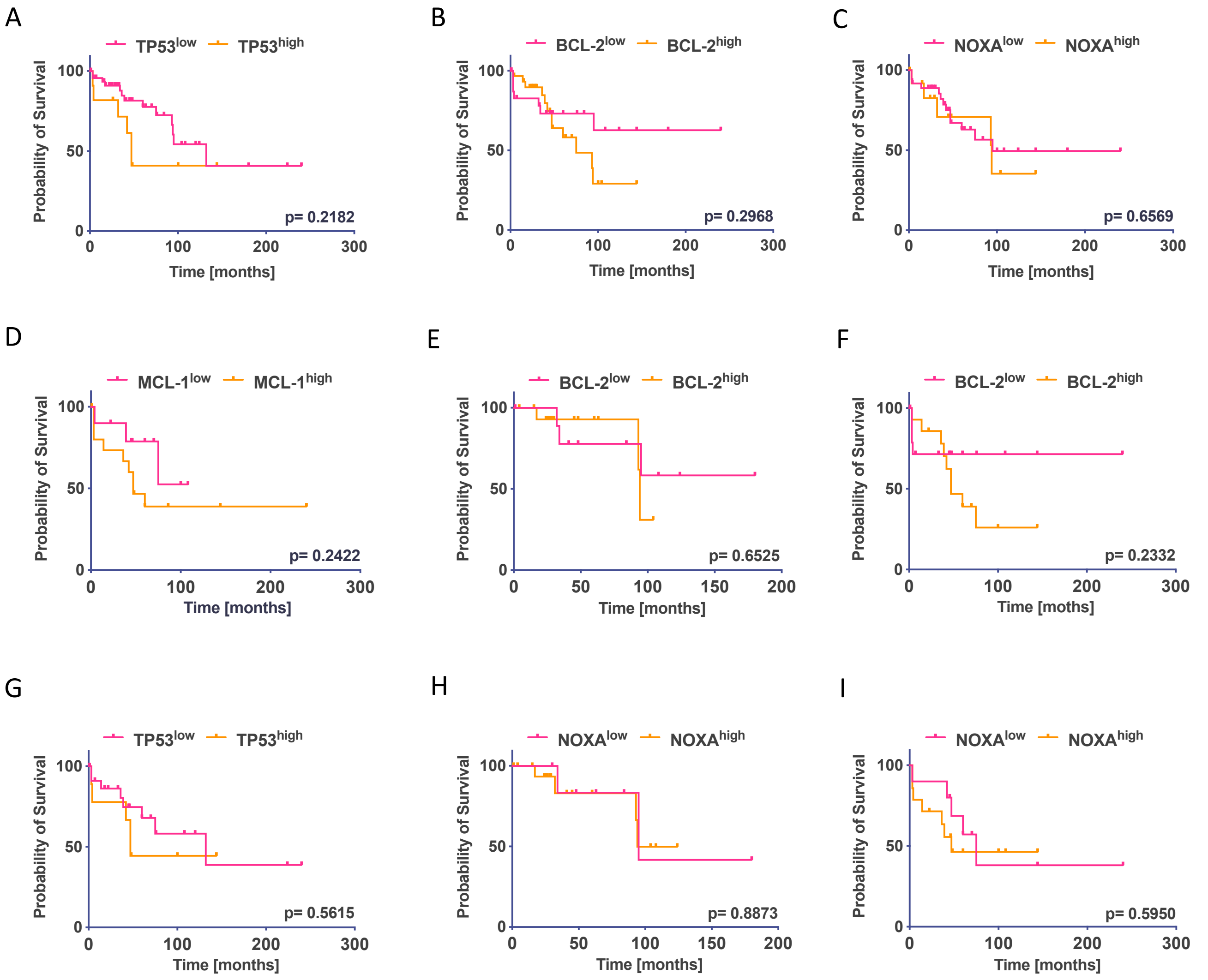
A
TP53low TP53high
Probability of Survival
Time [months]
p= 0.2182
B
BCL-2low BCL-2high
Probability of Survival
Time [months]
p= 0.2968
C
NOXAlow NOXAhigh
Probability of Survival
Time [months]
p= 0.6569
D
MCL-1low MCL-1high
Probability of Survival
Time [months]
p= 0.2422
E
BCL-2low BCL-2high
Probability of Survival
Time [months]
p= 0.6525
F
BCL-2low BCL-2high
Probability of Survival
Time [moths]
p= 0.2332
G
TP53low TP53high
Probability of Survival
Time [months]
p= 0.5615
H
NOXAlow NOXAhigh
Probability of Survival
Time [months]
p= 0.8873
I
NOXAlow NOXAhigh
Probability of Survival
Time [months]
p= 0.5950

Figure S2

A

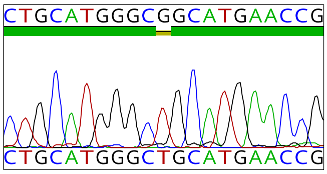

c.738G>T, p.M246I

B

	IC50 [μM]	
	1889c	HCC15
Cisplatin	5.0	17.3
Etoposide	1.6	8.4
Sutent	11.8	13.9
Sorafenib	10.7	8.8

C

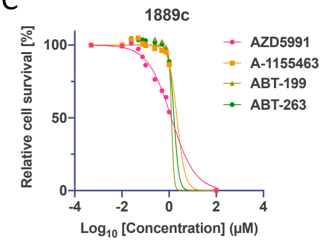

D

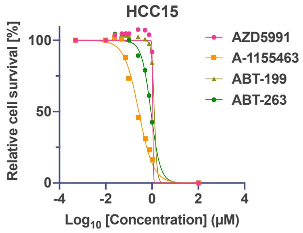

E

	IC50 [μM]	
	1889c	HCC15
AZD5991 (MCL-1)	1.2	1.1
A-1155463 (BCL-xL)	2.2	0.3
ABT-199 (BCL-2)	1.3	1.3
ABT-263 (BCL-2, BCL-xL, BCL-w)	1.6	0.9

F

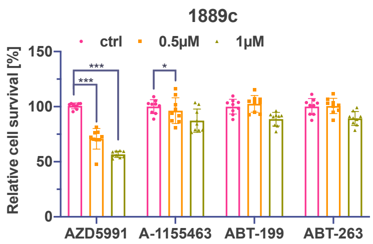

G

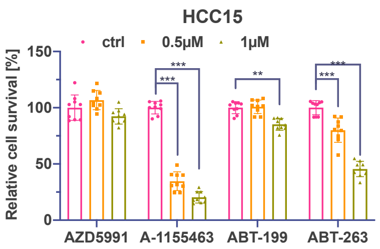

H

		IC50 [μM]
AZD5991 (MCL-1)		1.2
+ A-1155463 (BCL-xL)	+ 0.01μM	0.14
	+ 0.025μM	0.1
	+ 0.05μM	0.08
+ ABT-199 (BCL-2)	+ 0.01μM	1.2
	+ 0.025μM	1.2
	+ 0.05μM	1.4
+ ABT-263 (BCL-2, BCL-xL, BCL-w)	+ 0.1μM	0.14
	+ 0.25μM	0.08
	+ 0.5μM	0.05

I

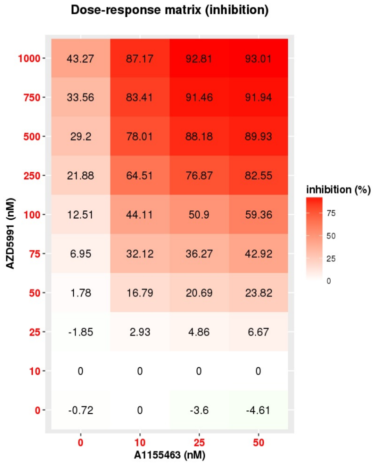

J

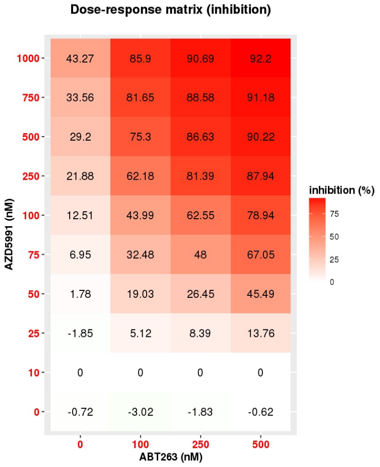

K

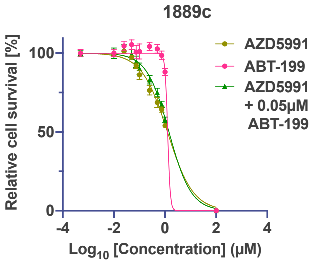

L

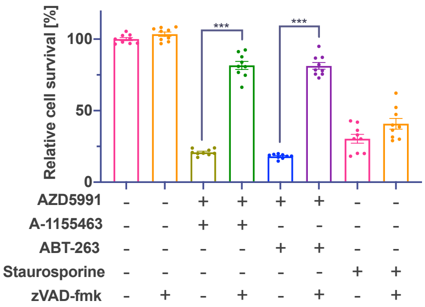

M

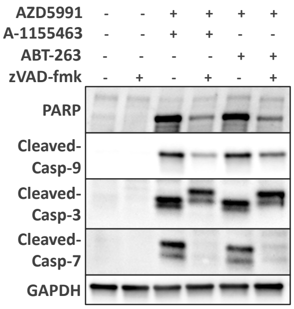

Figure S3

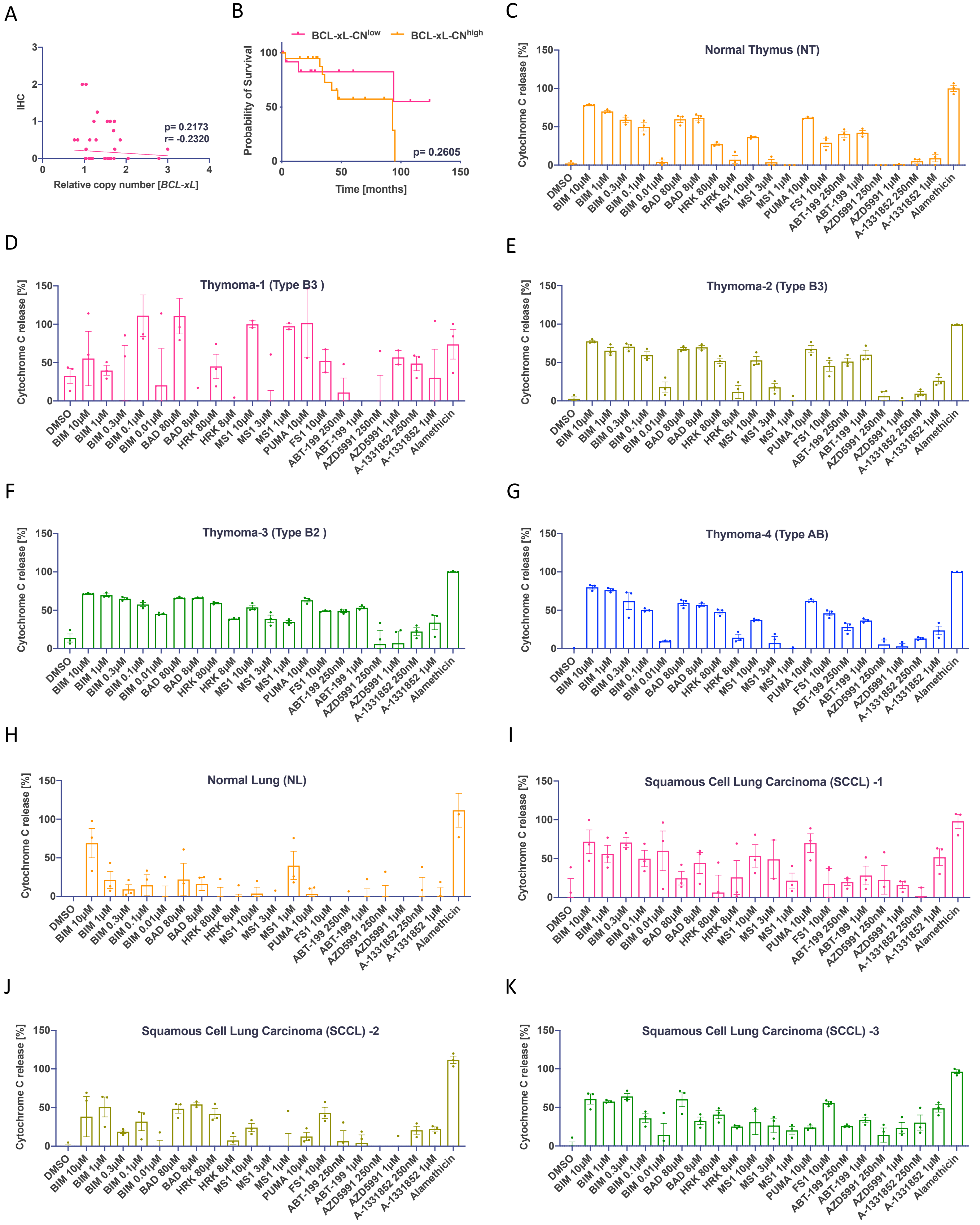

A
IHC
Relative copy number [BCL-xL]
p= 0.2173
r= -0.2320
B
BCL-xL-CN^low
BCL-xL-CN^high
Probability of Survival
Time [months]
p= 0.2605
C
Normal Thymus (NT)
Cytochrome C release [%]
DMSO
BIM 10µM
BIM 1µM
BIM 0.3µM
BIM 0.1µM
BIM 0.01µM
BAD 80µM
BAD 8µM
HRK 80µM
HRK 8µM
MS1 10µM
MS1 3µM
MS1 1µM
PUMA 10µM
FS1 10µM
ABT-199 250nM
ABT-199 1µM
AZD5991 250nM
AZD5991 1µM
A-1331852 250nM
A-1331852 1µM
Alamethicin
D
Thymoma-1 (Type B3)
E
Thymoma-2 (Type B3)
F
Thymoma-3 (Type B2)
G
Thymoma-4 (Type AB)
H
Normal Lung (NL)
I
Squamous Cell Lung Carcinoma (SCCL) -1
J
Squamous Cell Lung Carcinoma (SCCL) -2
K
Squamous Cell Lung Carcinoma (SCCL) -3

Table S1: Clinicopathological parameters of primary patient tissue samples.

Tissue	Masaoka-Koga	pT	N	L	V	R	Grading
Normal Thymus (NT)	-	-	-	-	-	-	-
Type B3 Thymoma (TH-1)	II	pT1a	N0	-	-	R0	-
Type B3 Thymoma (TH-2)	IIa	pT1a	N0	L0	V0	R0	-
Type B2 Thymoma (TH-3)	IIa	pT1a	N1	L0	V0	R0	-
Type AB Thymoma (TH-4)	I	pT1a	-	L0	V0	R0	-
Normal Lung (NL)	-	-	-	-	-	-	-
Squamous Cell Lung Carcinoma (SCCL-1)	-	pT4	N1	-	-	R0	G2
Squamous Cell Lung Carcinoma (SCCL-2)	-	pT2b	N0	L0	V0	R0	G3
Squamous Cell Lung Carcinoma (SCCL-3)	-	pT3	N0	-	V1	R0	G3

Table S2: Antibodies and chemical compounds.

Antibodies	Cat.No.	Western Blot Dilution	IHC Dilution
Primary – Cell Signaling			
BAK	#12105	1:1000	x
BAX	#2774	1:1000	x
BCL-2	#2872	1:1000	Ready to use (Dako)
BCL-xL	#2764	1:1000	1:2000
BIM	#2933	1:1000	x
Caspase-3	#9662	1:1000	x
Caspase-7	#9492	1:1000	x
Caspase-9	#9508	1:1000	x
Cleaved Caspase-3	#9664	1:1000	x
Cleaved Caspase-7	#8438	1:1000	x
Cleaved Caspase-9	#7237	1:1000	x
Cleaved PARP	#5625	1:1000	x
GAPDH	#2118	1:5000	x
MCL-1	#4572	1:1000	1:200
NOXA	#14766	1:1000	1:200
PARP	#9542	1:1000	x
Primary – Santa cruz			
TP53	sc-126	1:1000	Ready to use (Dako)
Secondary (all Sigma-Aldrich)			
Polyclonal Goat anti-rabbit	P0260	1:10000	x
Polyclonal Rabbit anti-mouse	P0448	1:10000	x

Compounds (all Selleckchem)	Cat.No.
A-1155463	#S7800
ABT-199 (Venetoclax)	#S8048
ABT-263 (Navitoclax)	#S1001
AZD-5991	#S8643
Cisplatin	#S1166
Etoposide	#S1225
Sorafenib	#S7397
Sunitinib	#S1042
zVAD.fmk	#S7023

Table S3: Primer sequences, plasmids and siRNAs.

Primer	Sequence
3p2-for	5’-CACAGAGTAAAGGCCCCGTGACTTT-3’
3p2-rev	5’-ACAGAAGGGCCAGAAAGAACCGAAC-3’
BCL-xL-for1	5’-TGTGCTTCAGAGATCAGGCA-3’
BCL-xL-rev1	5’-GGTCTGTGGGTCATTGATGC-3’
BCL-xL-for2	5’-GTTACCCTCTCTCCTCTGCC-3’
BCL-xL-rev2	5’-GAGGGAGGGATAAGGAAGCC-3’
MCL-1-for1	5’-GTCCCCAACTATGCCCTCTT-3’
MCL-1-rev1	5’-GTGCTTCCCTGAGACCTGAT-3’
MCL-1-for2	5’-AGTGGTATGGCGGTCTTGAA-3‘
MCL-1-rev2	5’-TCCATTAAAACAGGCACCGC-3’
**Plasmids (all OriGene Technologies)**	**Cat.No.**
NOXA (PMAIP1) (NM_021127) Human Tagged ORF clone	RC202071
pCMV6-Entry Mammalian Expression Vectore	PS100001
**siRNAs (all Qiagen)**	**Cat.No.**
Hs_BCL2L1_2	#SI00023191
Hs_BCL2L1_10	#SI03112018
Hs_MCL1_6	#SI02781205
Hs_MCL1_8	#SI03069003
Neg. ctrl. siRNA	#1027281
