# Supplementary material for: Functional apoptosis profiling identifies MCL-1 and BCL-xL as prognostic markers and therapeutic targets in advanced thymomas and thymic carcinomas
Source: BMC Med. 2021 Nov 16;19:300. doi: 10.1186/s12916-021-02158-3 (PMC8594228; doi:10.1186/s12916-021-02158-3)
Supplement: Supplementary file 2 — Additional file 2. Unprocessed Fig. 2C. [file 12916_2021_2158_MOESM2_ESM.pdf]

Additional file 2: Unprocessed figure 2C

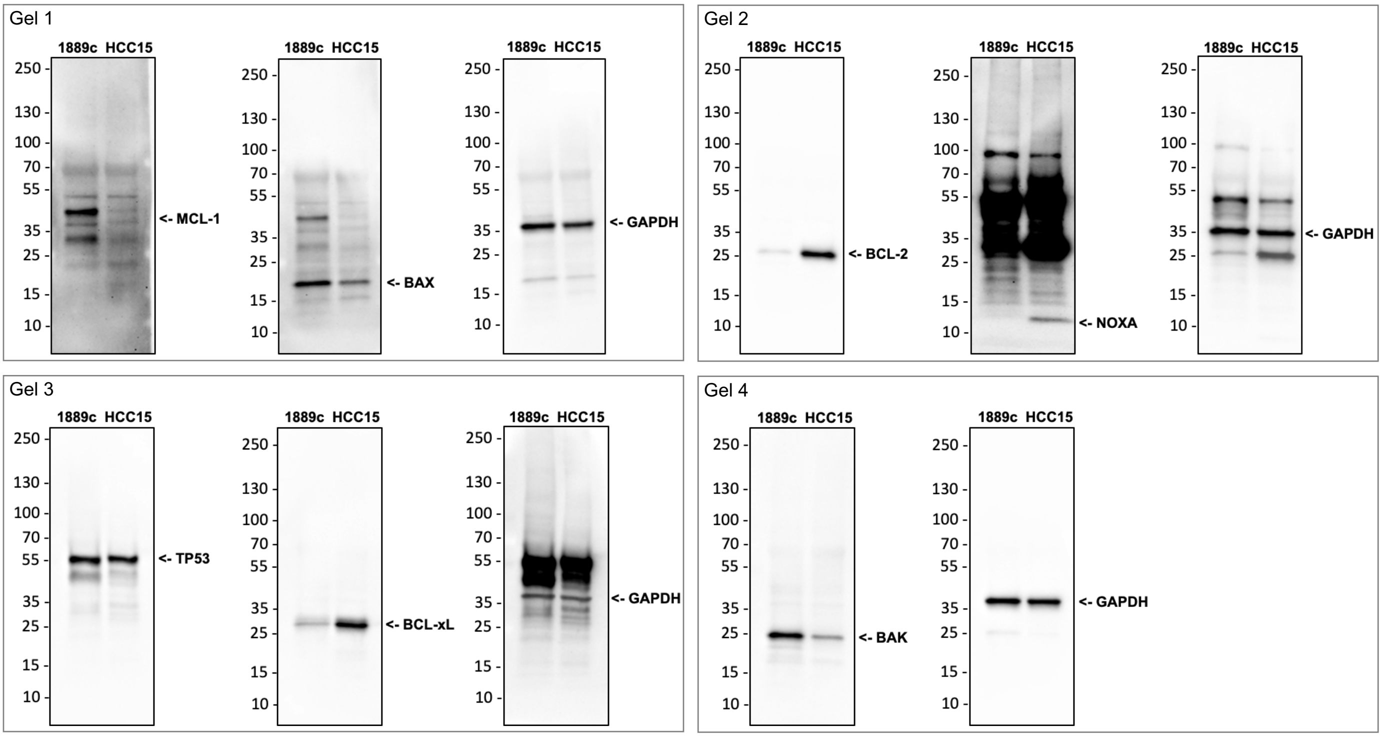

Legend of Figure 2C: Analysis of BCL-2 family proteins in the chemo-resistant 1889c reveals an MCL-1 dependent apoptotic priming. Western Blot analysis of TP53, MCL-1, BCL-2, BCL-xL, BAX, BAK, and NOXA in 1889c and HCC15.

Additional file 2: Unprocessed figure 2H

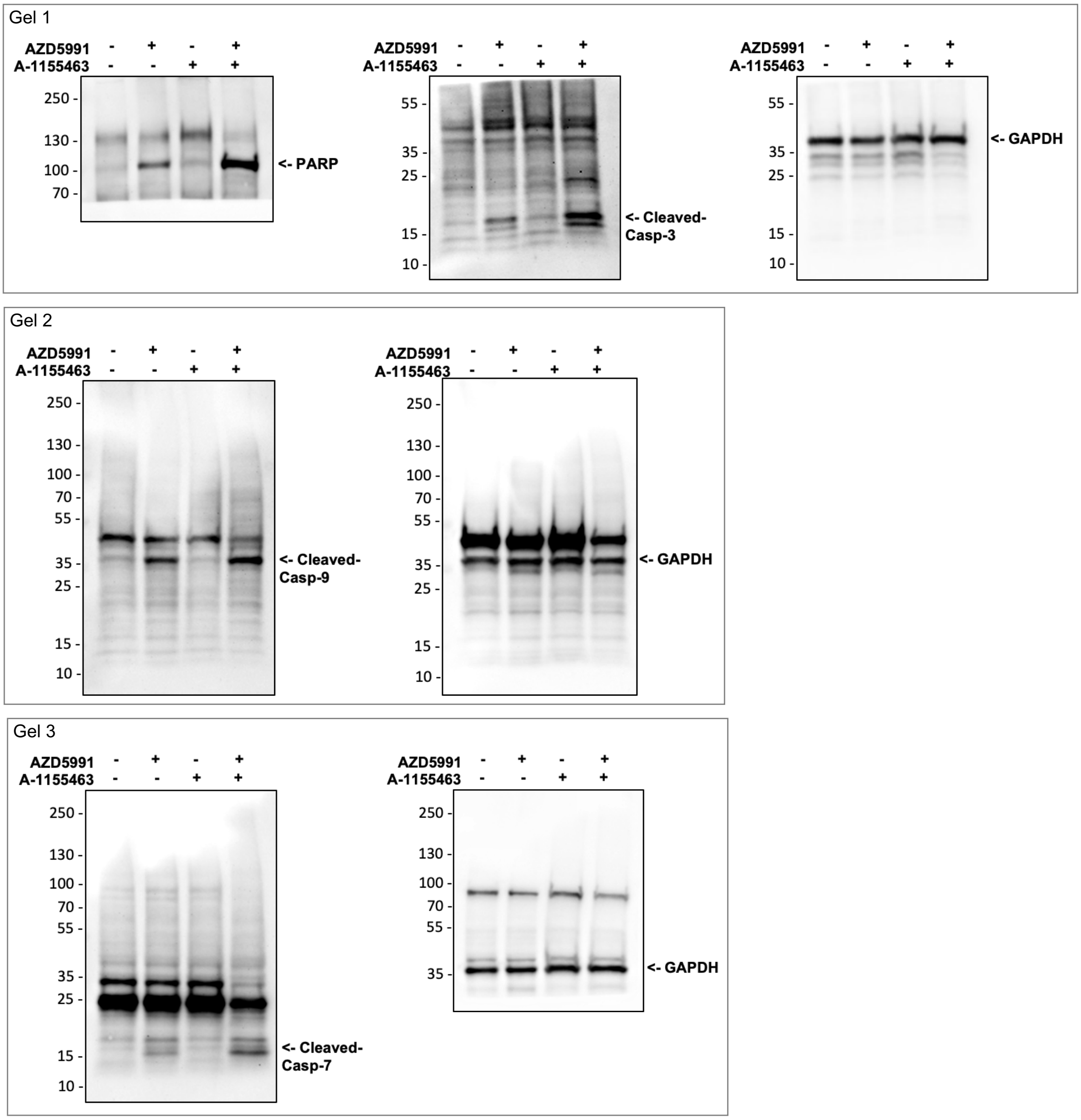

Legend of Figure 2H: Analysis of BCL-2 family proteins in the chemo-resistant 1889c reveals an MCL-1 dependent apoptotic priming. Western Blot analysis of PARP cleavage and caspase activation of 1889c after a 6h single treatment with AZD5991 (0.5µM) and A-1155463 (0.05µM) and the combination of AZD5991 and A-1155463.

Additional file 2: Unprocessed figure 2L

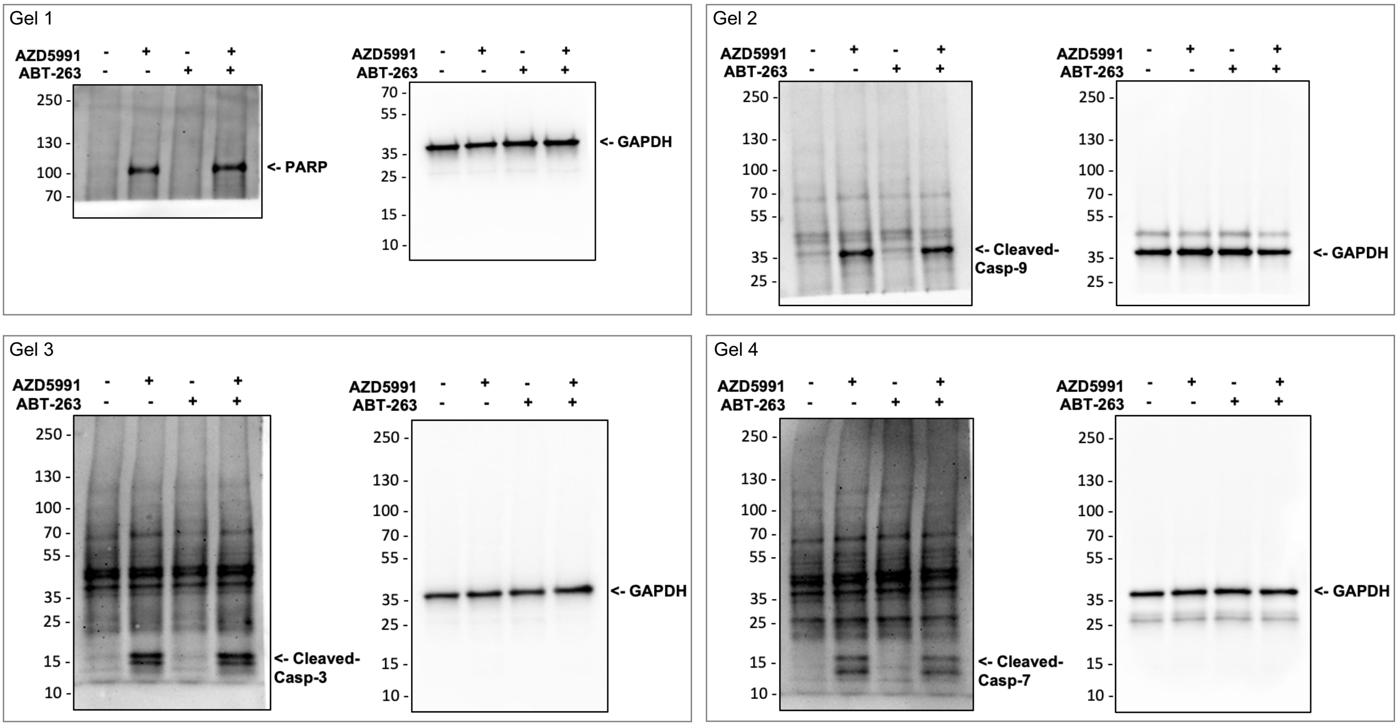

Legend of Figure 2L: Analysis of BCL-2 family proteins in the chemo-resistant 1889c reveals an MCL-1 dependent apoptotic priming. Analysis of PARP cleavage and caspase activation of 1889c after a 6h single treatment with AZD5991 (0.5µM) and ABT-263 (0.5µM) and the combination of AZD5991 and ABT-263 by Western Blot.

Additional file 2: Unprocessed figure 3A

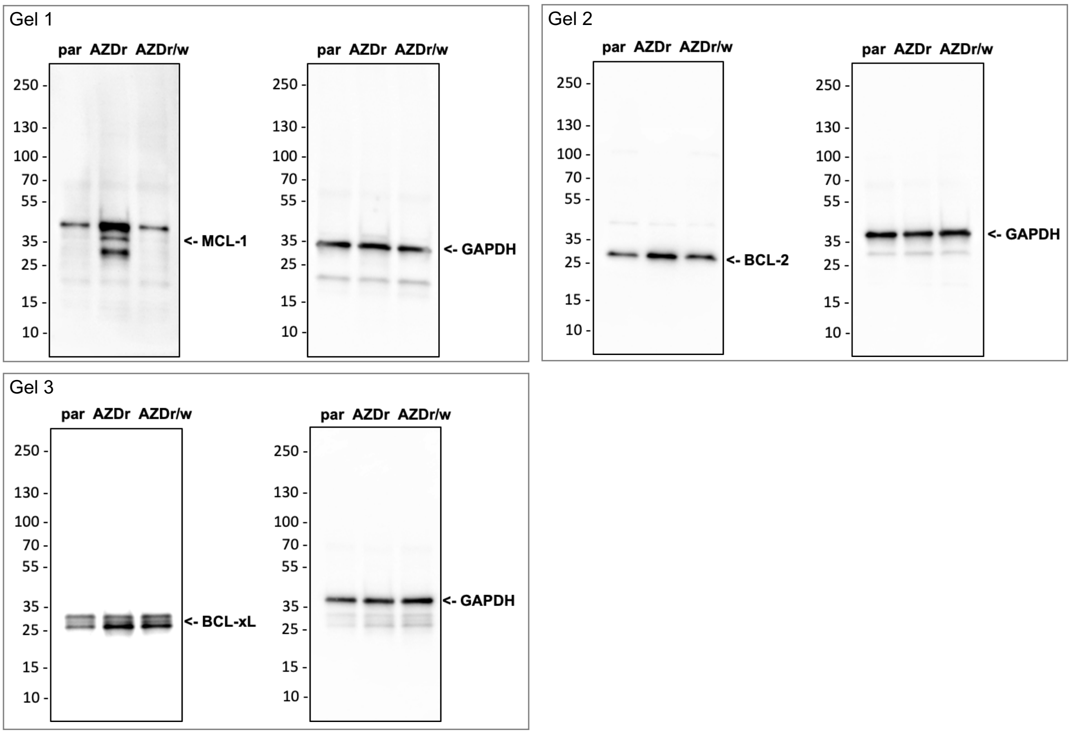

Legend of Figure 3A: AZD5991 resistance induction, siRNA knockdown, and IP of BIM show a synergistic effect of MCL-1 and BCL-xL. Western Blot showing upregulation of MCL-1 and BCL-xL in AZD5991 resistant 1889c (1889c-AZDr).

Additional file 2: Unprocessed figure 3E

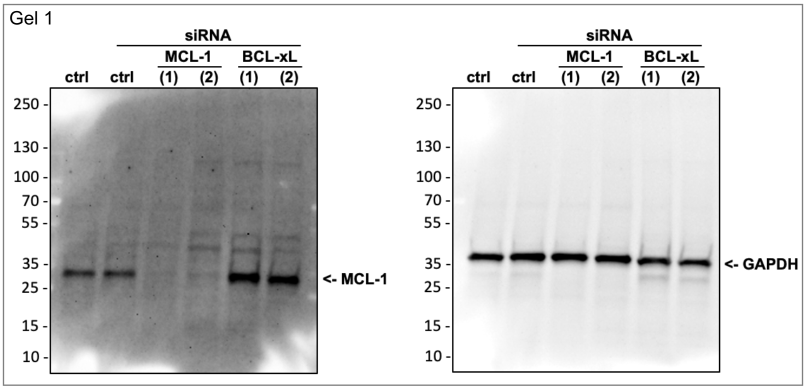

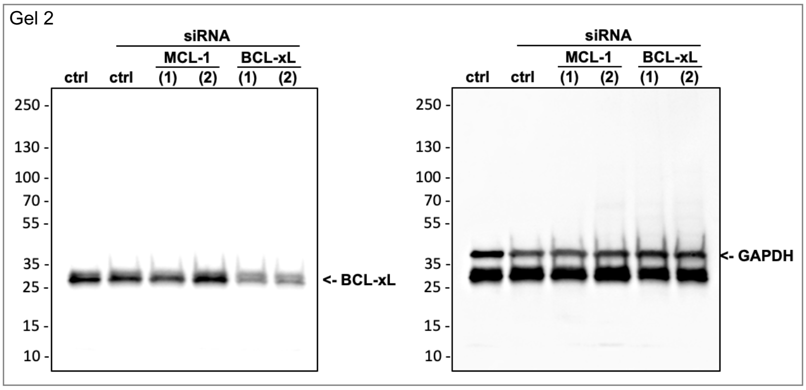

Legend of Figure 3E: AZD5991 resistance induction, siRNA knockdown, and IP of BIM show a synergistic effect of MCL-1 and BCL-xL. Western Blot analysis of 1889c after MCL-1 and BCL-xL knockdown with two individual siRNAs.

Additional file 2: Unprocessed figure 3I

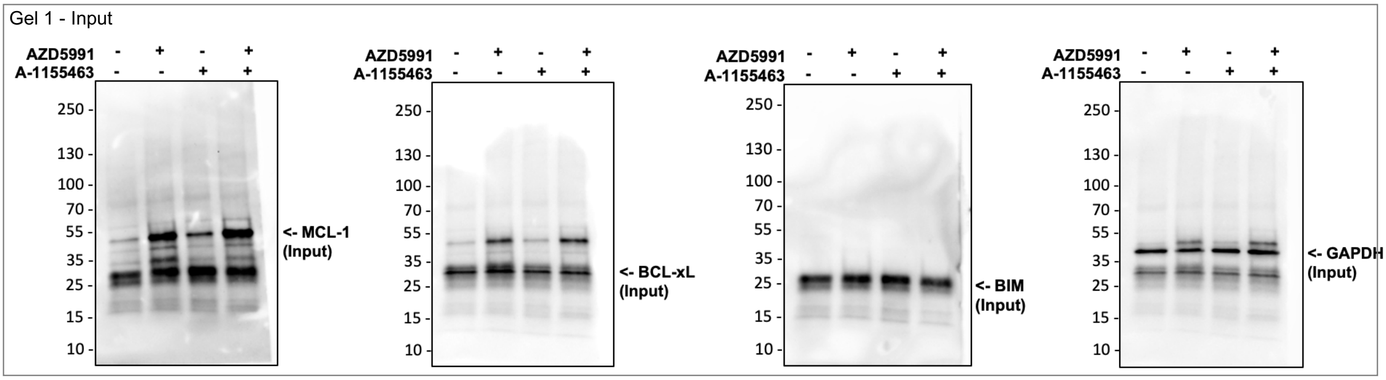

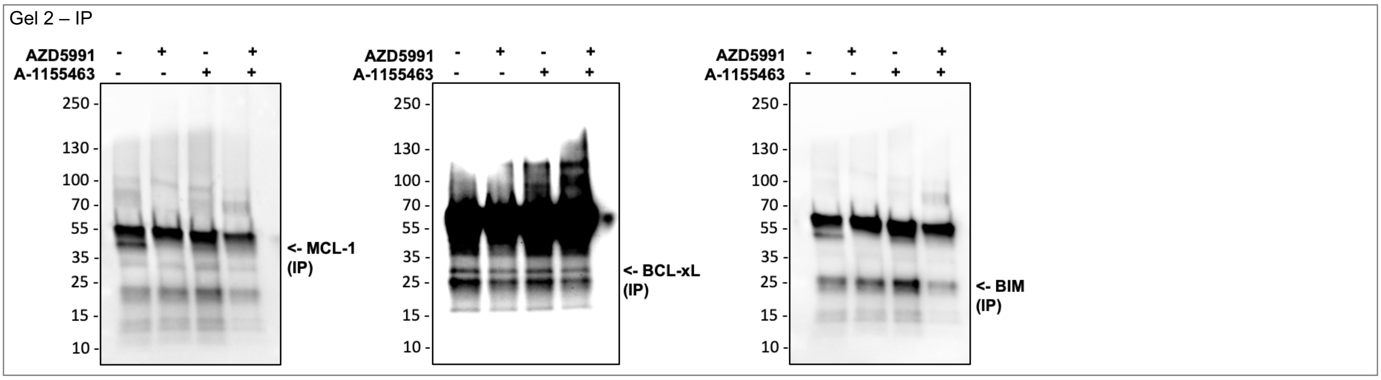

Legend of Figure 3I: AZD5991 resistance induction, siRNA knockdown, and IP of BIM show a synergistic effect of MCL-1 and BCL-xL. Redistribution of BIM in 1889c cells after 4h treatment with AZD5991 (0.1μM), A-1155463 (0.1μM), or a combination of both (Western Blot after BIM IP).

Additional file 2: Unprocessed ofigure 4B

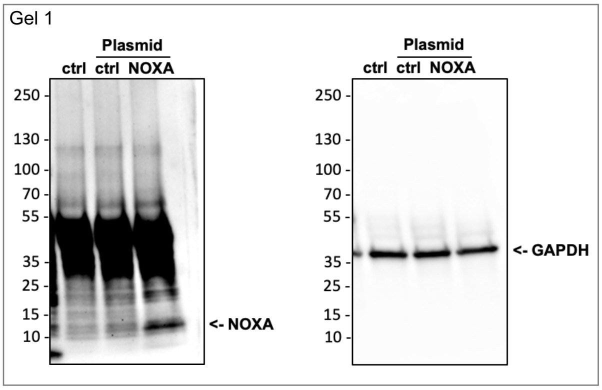

Legend of Figure 4B: Correlation between IHC staining of MCL-1 and BCL-xL and OS in NOXAlow TH and TC and re-expression of NOXA in 1889c. Western Blot analysis of 1889c cells transfected with control or NOXA plasmid.

Additional file 2: Unprocessed figure 5E

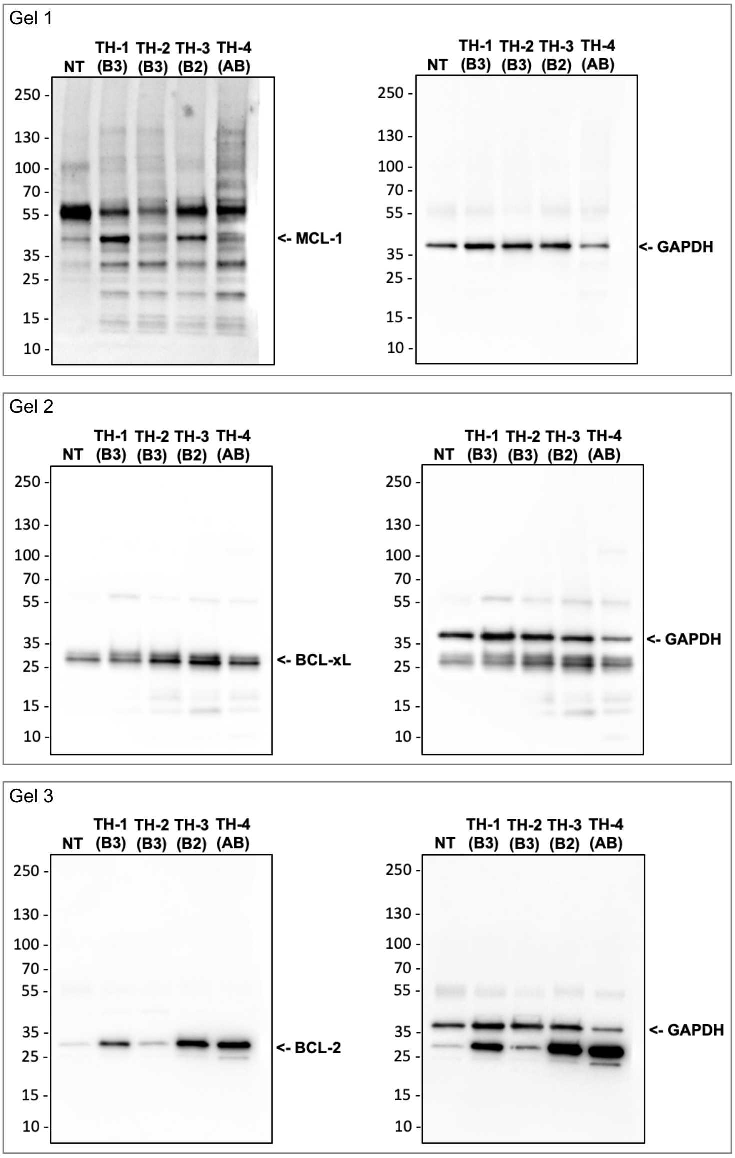

Legend of Figure 5E: BH3 profiling of primary patient tissue samples predicts apoptotic priming and therapy response to BH3 mimetics. Western Blot analysis of MCL-1, BCL-2, and BCL-xL expression of a healthy thymus (NT), two type B3 (TH-1 and 2), a type B2 (TH-3), and a type AB (TH-4) TH.

Additional file 2: Unprocessed figure 5H

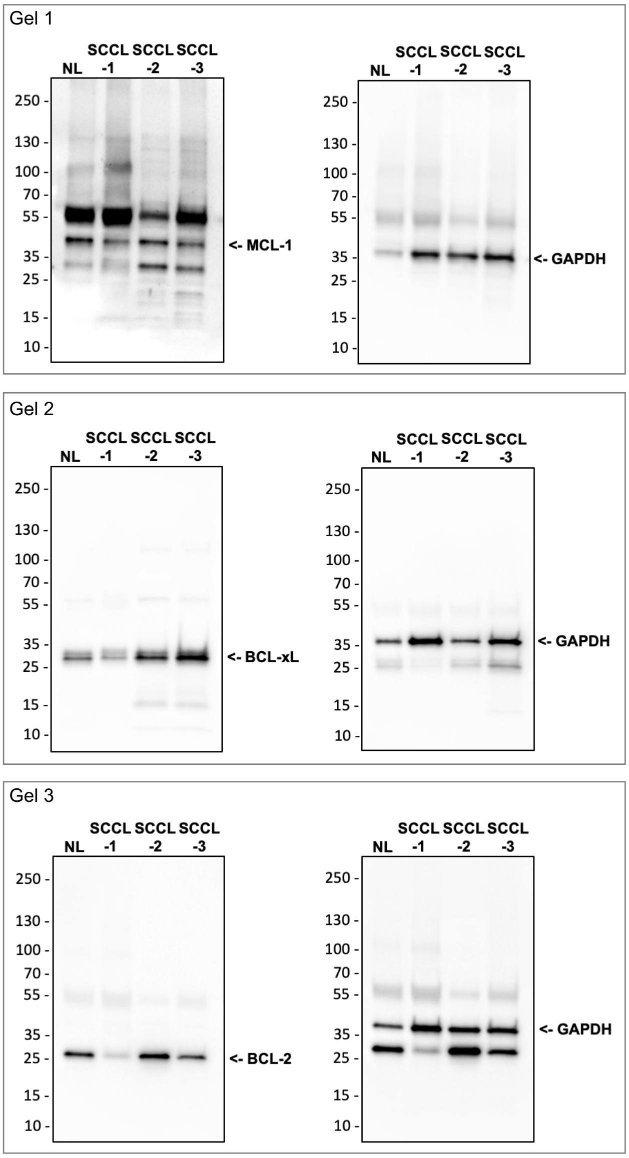

Legend of Figure 5E: BH3 profiling of primary patient tissue samples predicts apoptotic priming and therapy response to BH3 mimetics. Western Blot analysis of a healthy lung (NL) and three SCCL samples.

Additional file 2: Unprocessed supplementary figure 2M

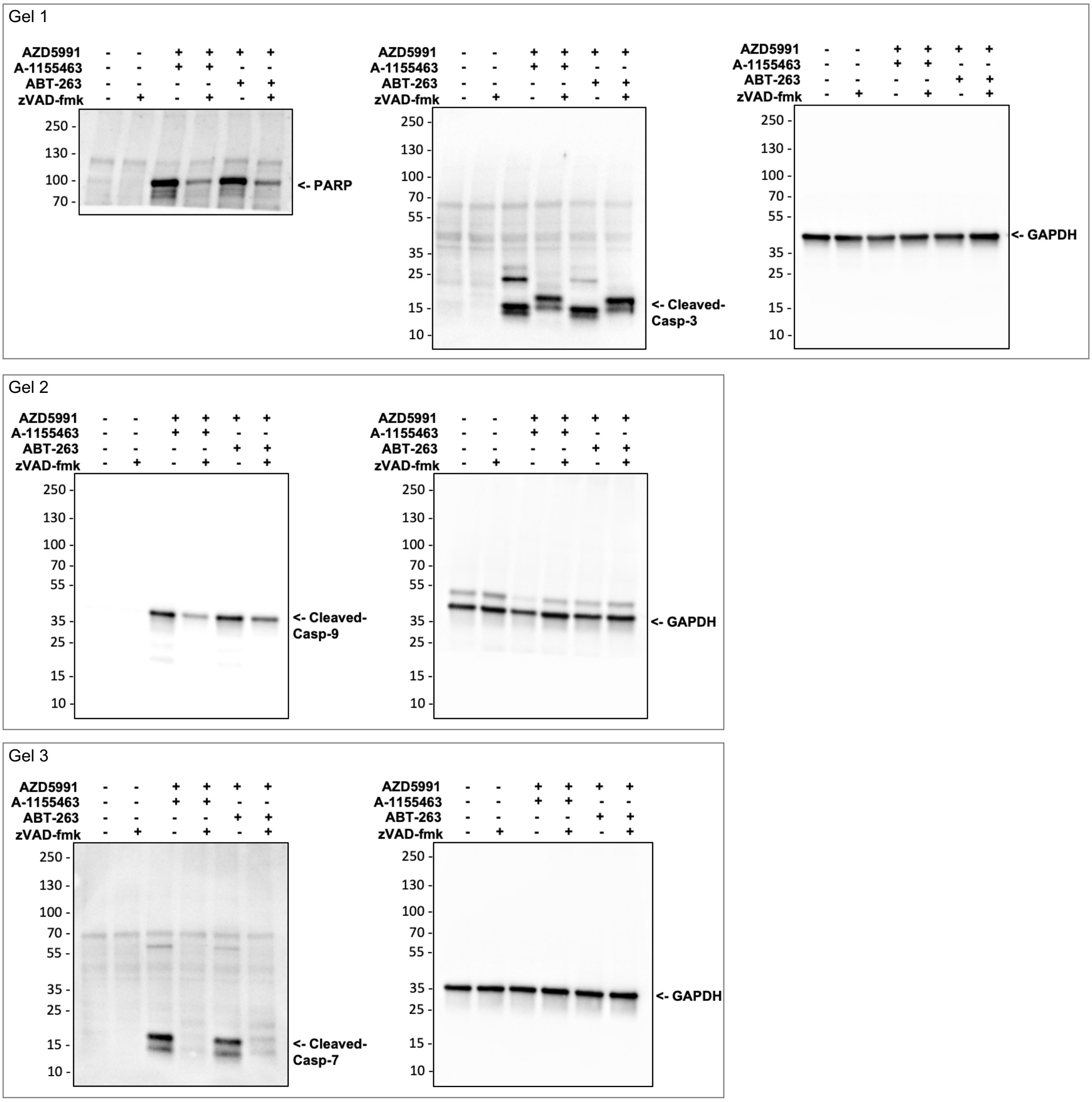

Legend of Figure 2M: Analysis of BCL-2 family members and specific inhibitors in 1889c and HCC15. Western Blot analysis of 1889c cells treated for 24h with AZD5991 (0.5µM), A-1155463 (0.05µM), ABT-263 (0.5µM), and staurosporine (0.25µM) alone or after 3h pretreatment with the pan-caspase inhibitor zVAD-fmk (100µM).
